# Supplementary material for: Pasa: leveraging population pangenome graph to scaffold prokaryote genome assemblies
Source: Nucleic Acids Res. 2023 Dec 12;52(3):e15. doi: 10.1093/nar/gkad1170 (PMC10853769; doi:10.1093/nar/gkad1170)
Supplement: gkad1170_supplemental_file [file gkad1170_supplemental_file.pdf]

Supplementary Material to Pasa: Leveraging population pangenome graph to scaffold prokaryote genome assemblies

Van Hoan Do[1, *], Son Hoang Nguyen[2], Duc Quang Le[3], Tam Thi Nguyen[4], Canh Hao Nguyen[6], Tho Huu Ho[7,8] , Nam Sy Vo[9], Trang Nguyen[2], Hoang Anh Nguyen[2], and Minh Duc Cao[2, *]

[1]Center for Applied Mathematics and Informatics, Le Quy Don Technical University, Hanoi, Vietnam; [2]AMROMICS JSC, Nghe An, Vietnam ; [3]Faculty of IT, Hanoi University of Civil Engineering, Hanoi, Vietnam; [4]Oxford University Clinical Research Unit, Hanoi, Vietnam; [5]Bioinformatics Center, Institute for Chemical Research, Kyoto University, Japan; [6]Department of Medical Microbiology, The 103 Military Hospital, Vietnam Military Medical University, Hanoi, Vietnam; [7]Department of Genomics & Cytogenetics, Institute of Biomedicine & Pharmacy, Vietnam Military Medical University, Hanoi, Vietnam; [8]Center for Biomedical Informatics, Vingroup Big Data Institute, Hanoi, Vietnam

[*]To whom correspondence should be addressed. E-mails:hoandv@lqdtu.edu.vn; minhduc.cao@gmail.com

1. Supplementary Figures

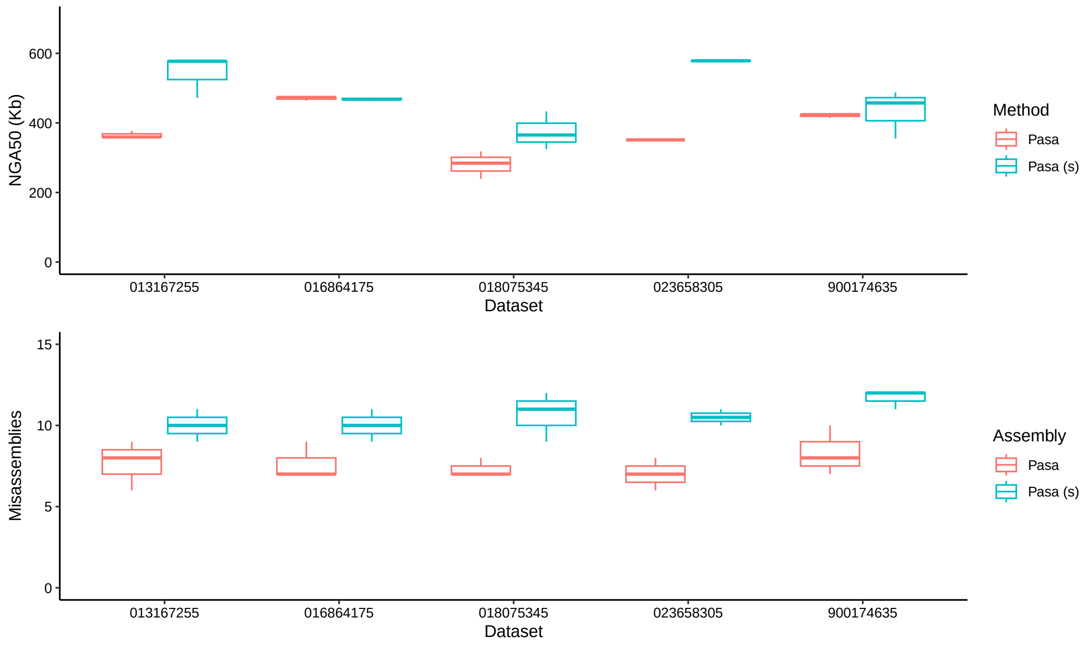

Figure S1: The performance of Pasa under the influence of the order of the reference genomes in "Construction of the pangenome graph". For each of the five *E. coli.* data sets, the box plot shows minimum, maximum, median, and first and third quartiles of NGA50 scores (top) and Misassemblies (bottom) achieved by Pasa on the 10 times randomly shuffling the order of the reference genomes.

2. Supplementary Tables

Species	Accession
Klebsilla pneumoniae	https://www.ebi.ac.uk/ena/browser/view/GCA_001705385.1
Klebsilla pneumoniae	https://www.ebi.ac.uk/ena/browser/view/GCA_021390015.1
Klebsilla pneumoniae	https://www.ebi.ac.uk/ena/browser/view/GCA_021249265.1
Klebsilla pneumoniae	https://www.ebi.ac.uk/ena/browser/view/GCA_021166135.1
Klebsilla pneumoniae	https://www.ebi.ac.uk/ena/browser/view/GCA_021166095.1
Klebsilla pneumoniae	https://www.ebi.ac.uk/ena/browser/view/GCA_004322955.1
Klebsilla pneumoniae	https://www.ebi.ac.uk/ena/browser/view/GCA_001521895.1
Klebsilla pneumoniae	https://www.ebi.ac.uk/ena/browser/view/GCA_001456135.1
Klebsilla pneumoniae	https://www.ebi.ac.uk/ena/browser/view/GCA_001307175.1
Klebsilla pneumoniae	https://www.ebi.ac.uk/ena/browser/view/GCA_000814805.1
Escherichia coli	https://www.ebi.ac.uk/ena/browser/view/GCA_900174635.1
Escherichia coli	https://www.ebi.ac.uk/ena/browser/view/GCA_023658305.1
Escherichia coli	https://www.ebi.ac.uk/ena/browser/view/GCA_022918835.1
Escherichia coli	https://www.ebi.ac.uk/ena/browser/view/GCA_020526805.1
Escherichia coli	https://www.ebi.ac.uk/ena/browser/view/GCA_018075345.1
Escherichia coli	https://www.ebi.ac.uk/ena/browser/view/GCA_016864175.1
Escherichia coli	https://www.ebi.ac.uk/ena/browser/view/GCA_015571535.1
Escherichia coli	https://www.ebi.ac.uk/ena/browser/view/GCA_014295255.1
Escherichia coli	https://www.ebi.ac.uk/ena/browser/view/GCA_014169015.1
Escherichia coli	https://www.ebi.ac.uk/ena/browser/view/GCA_013167255.1
Streptococcus pneumoniae	https://www.ebi.ac.uk/ena/browser/view/GCA_022318405.1
Streptococcus pneumoniae	https://www.ebi.ac.uk/ena/browser/view/GCA_022075545.1
Streptococcus pneumoniae	https://www.ebi.ac.uk/ena/browser/view/GCA_022070425.1
Streptococcus pneumoniae	https://www.ebi.ac.uk/ena/browser/view/GCA_022069545.1
Streptococcus pneumoniae	https://www.ebi.ac.uk/ena/browser/view/GCA_022069445.1
Streptococcus pneumoniae	https://www.ebi.ac.uk/ena/browser/view/GCA_022068405.1
Streptococcus pneumoniae	https://www.ebi.ac.uk/ena/browser/view/GCA_019456615.1
Streptococcus pneumoniae	https://www.ebi.ac.uk/ena/browser/view/GCA_008253725.1
Streptococcus pneumoniae	https://www.ebi.ac.uk/ena/browser/view/GCA_003966525.1
Streptococcus pneumoniae	https://www.ebi.ac.uk/ena/browser/view/GCA_000251085.1

Table S1: Accessions of the isolates used in the study

3. Supplementary Methods

3.1. Construction of the pangenome using PanTA

PanTA is developed with the aim to build the pangenome of a large collection of genomes, and to add a set of new genomes to an existing pangenome without rebuilding the accumulated pangenome from scratch. The core of the pipeline is the clustering of all genes in the collection into gene clusters, that represent the gene families in the collection. PanTA employs a similar clustering strategy as most pangenome methods such as Roary [1], PIRATE [2] and Panaroo [3]. It first runs CD-HIT [4] to group similar protein sequences together, and essentially reduces the set of all protein sequences to a smaller set of representative sequences from the groups. The default identity threshold for CD-HIT grouping is 98% and the value can be adjusted by users. PanTA then performs an all-against-all alignment of the representative sequences with diamond [5]. The resulting pairwise alignments are filtered to retain those that pass certain thresholds of sequence identity (default at 70%), alignment length ratios, and length difference ratios. These alignments are inputted into Markov clustering (MCL) [6] that clusters the representative sequences into homologous groups of genes. Each protein sequence is then assigned to the gene cluster its representative sequence belongs to.

PanTA can run in progressive mode where it adds new genomes into an existing pangenome without rebuilding the pangenome from scratch. In this mode, PanTA uses CD-HIT-2D, a tool in CD-HIT suite [4] to match new protein sequences extracted from the new samples to the representative sequences from the existing groups. The protein sequences that are matched are assigned to the existing groups and by proxy, to existing gene clusters while unmatched sequences are subject to CD-HIT to create new groups. Similarly, during the all-against-all alignment step, PanTA first performs alignment of the representative sequences of the new groups against the representative sequences of the existing groups. It also runs the all-against-all alignment of the new representative sequences. The two sets of alignments after filtering are combined and are subject to MCL clustering. With the strategy, PanTA reduces the number of sequences in the grouping and alignment steps which are the most resource-intensive steps of the whole pipeline, and hence significantly speeds up the process.

3.2. Construction of the pangenome graph

Pasa builds the pangenome graph of the species from the genome assemblies of a collection of isolates. During pangenome graph construction, the input assemblies are annotated using Prokka [7] with the command line "prokka –outdir prokkaout –prefix mygenome contigs.fa" to get an annotation file in "gff" format for each genome assembly. Pasa then runs PanTA on the population gff files to obtain gene clusters. Here each cluster represents an orthologous or paralogous group of genes. PanTA groups the genes based on their sequence similarity and outputs a sequence of genes for each chrosomome or plasmid in the reference genomes. We use PanTA with default parameters "panta main -o output -g prokkaout/*.gff" (identity = 0.7, e-value = 1e-6).

Next, Pasa orients the gene-level genomes obtained by PanTA such that the number of common consecutive gene pairs between two genomes are maximal. The orientations of the gene-level genomes are achieved by the following heuristic procedure: The algorithm starts with the first genome, and its orientation is arbitrary. Next, Pasa finds an orientation of the second genome that has the largest number of common pairs of consecutive genes with the first genome. Similarly, Pasa finds an orientation of the third genome that shares the largest number of common pairs of consecutive genes with the first two genomes, and the procedure is repeated for the remaining genomes. For example, suppose that $(A, B, C), (C, B, A), (D, B, A)$ are three gene sequences produced by PanTA, where A, B, C, D are genes. By this procedure, Pasa will flip the orientation of the second genome to (A, B, C) that shares two consecutive pairs (A, B) and (B, C) with the first genome. Similarly, the orientation of the third genome is flipped to (A, B, D). Pasa then constructs a directed graph $G = (V, E, w)$ with weighted edges w, where nodes V represent clusters of genes, two nodes are connected by an edge (in E) if they are adjacent in any genome from the population, and the edge weight accounts for the number of times two nodes are adjacent in the oriented genomes. With the above three genomes, the pangenome graph is given by $G = (V, E, w)$, where $V = \{A, B, C, D\}, E = \{(A, B), (B, C), (B, D)\}$, and weights $w(A, B) = 3, w(B, C) = 2, w(B, D) = 1$.

Pasa then remove all edges whose weights are smaller than 20% the number of the reference genomes. All algorithms and data structures in Pasa are implemented in Python API package: https://github.com/amromics/pasa. The graph construction and edge filtering are provided with the "construct_graph()" function.

3.3. Construction of contigs overlap graph of the input genome

The input genome is sequenced by NGS platforms and provided as *e.g.* paired-end reads in FASTQ format. We then use SPAdes/Shovill to generate the short-reads assembly of contigs and their underlying assembly graph. For example:

```
# spades.py --isolate -1 R1.fq.gz -2 R2.fq.gz
```

Based on the *de Bruijn* assembly graph from SPAdes, Pasa builds a sequence overlap graph of all final contigs, or the *contigs graph* for short throughout the scope of this article to distinguish it from the other graph structures. The SPAdes assembly graph is saved in a FASTG file, namely `assembly_graph.fastg` by default. It also can be made available in GFA format if using recent versions of SPAdes. However, we use the former to support a wider range of this software versioning. The sequences in the FASTG file are edges from the assembly graph, also known as preliminary contigs before the repeat resolution. The final contigs are then constructed from the consequential repeat resolving step, for each of them comprises a unique path of preliminary contigs traversing this graph (`contigs.paths`). By combining information from these two files, we are able to construct the graph with contigs as vertices and their *k-mer* overlapping connections as edges.

Formally, Pasa builds a graph $\mathcal{G}_C = \{\mathcal{C}, \mathcal{V}_\mathcal{C}\}$ where $\mathcal{C} = \{c_1, c_2, \ldots, c_n\}$ is the set of the final contigs and $\mathcal{V}_\mathcal{C}$ is all possible k-overlap edges connecting them. From `assembly_graph.fastg`, we have the graph $\mathcal{G}_\mathcal{E} = \{E, V_E\}$ of preliminary contig sequences $E = \{e_i\}, i = 1 \ldots m$ and their connections $V_E = \{(e_i, e_j)\}$ for all e_i k-overlap with e_j. By investigating the file `contigs.paths`, we know how a final contig is made from a path of the preliminary sequences. For example, if we have $c_k = (e_{k_1}, e_{k_2}, \ldots, e_{k_p})$, $c_h = (e_{h_1}, e_{h_2}, \ldots, e_{h_q})$ then there is an edge $(c_k, c_h) \in V_C$ if and only if $(e_{k_p}, e_{h_1}) \in V_E$. In the case when intermediate files from SPAdes output are not given but only the final contigs (`contigs.fasta`), Pasa will scan for all possible overlaps between all pairs $(c_i, c_j) \in \mathcal{C}$. The scanning window for overlapping length is set to the range $[\frac{l_{min}-1}{2}, l_{min} - 1]$ by default, where l_{min} is the length of the shortest contig amongst $\mathcal{C}$.

3.4. Pangenome graph-based assembly model

Alignment of contigs in T to the pangenome graph The draft assembly of the target genome T is annotated with prokka with the same command line as above and is added into the pangenome graph by the add function in PanTA as follows: "panta add -c output -g prokkaout/target_genome.gff", where "ouput" is the output directory of PanTA and "prokkaout/target_genome.gff" is the location of the prokka annotated input genome.

Multiplicity estimation To estimate the multiplicity of contigs (copy number) in the target genome, Pasa uses the length and coverage information. In particular, the median coverage of the five largest contigs is the baseline (assume that it is D). Then for each contig x, its median read depth d_x is a good indicator of its multiplicity m_x, and $m_x = [d_x/D]$, which is the ratio of d_x and D, rounding to the nearest integer. The multiplicity estimation is obtained by the function "compute_multiplicity()" in Pasa Python API.

Matching scores Given a collection of contigs $\mathcal{C} = \{c_1, c_2, \ldots, c_n\}$, Pasa assigns a score to each pair of contigs in $\mathcal{C}$. The score between two contigs c_i and c_j (score(c_i, c_j)) is assigned to 0 if the shortest distance (in nucleotides) between the two contigs in the contigs graph is less than 5,000; otherwise it is assigned to $s(c_i, c_j)$, which is the ratio of the distance (between the terminal gene in c_i and a starting gene in c_j in the pangenome graph) and the number of genes between the two genes. If there is more than one path between two genes, Pasa takes the shortest one. The matching scores are implemented in the beginning of the function "join_contig()" in Pasa Python API.

Constrained maximum matching model Given the matching scores, Pasa employed the constrained maximum matching algorithm (see Algorithm 2 in the main paper) to find an arrangement of the contigs in the target genome. The algorithm is implemented in the end of the function "join_contig()".

Refinement To include un-aligned contigs and short contigs that have no gene, Pasa uses the contigs graph to insert these contigs into the above assemblies. The complete algorithm pipeline of Pasa as well as the refinement steps are implemented in the function "run_pangraph_pipeline()". The Pasa software is available at GitHub (https://github.com/amromics/pasa) under the open-source MIT license. The Pasa repository also includes all code necessary to reproduce the results of this manuscript.

References

[1] Page, A.J., Cummins, C.A., Hunt, M., Wong, V.K., Reuter, S., Holden, M.T., Fookes, M., Falush, D., Keane, J.A., Parkhill, J.: Roary: rapid large-scale prokaryote pan genome analysis. Bioinformatics **31**(22), 3691–3693 (2015)

[2] Bayliss, S.C., Thorpe, H.A., Coyle, N.M., Sheppard, S.K., Feil, E.J.: PIRATE: A fast and scalable pangenomics toolbox for clustering diverged orthologues in bacteria. GigaScience **8**(10), 1–9 (2019). doi:10.1093/gigascience/giz119

[3] Tonkin-Hill, G., MacAlasdair, N., Ruis, C., Weimann, A., Horesh, G., Lees, J.A., Gladstone, R.A., Lo, S., Beaudoin, C., Floto, R.A., *et al.*: Producing polished prokaryotic pangenomes with the panaroo pipeline. Genome biology **21**, 1–21 (2020)

[4] Li, W., Godzik, A.: Cd-hit: a fast program for clustering and comparing large sets of protein or nucleotide sequences. Bioinformatics **22**(13), 1658–1659 (2006). doi:10.1093/bioinformatics/btl158

[5] Buchfink, B., Xie, C., Huson, D.H.: Fast and sensitive protein alignment using DIAMOND. Nature Methods **12**(1), 59–60 (2015). doi:10.1038/nmeth.3176

[6] Enright, A.J.: An efficient algorithm for large-scale detection of protein families. Nucleic Acids Research **30**(7), 1575–1584 (2002). doi:10.1093/nar/30.7.1575

[7] Seemann, T.: Prokka: rapid prokaryotic genome annotation. Bioinformatics **30**(14), 2068–2069 (2014). doi:10.1093/bioinformatics/btu153
